# Adjuvant chemotherapy after curative D2 gastrectomy in Latin American patients with gastric cancer

**DOI:** 10.3332/ecancer.2022.1387

**Published:** 2022-05-12

**Authors:** Mariana Serrano, Jhajaira M Araujo, Cristian Pacheco, Jackeline Macetas, Mariella A Blum, Alfredo Carrato, Eloy Ruiz, Francisco Berrospi, Carlos Luque, Ivan Chavez, Eduardo Payet, Luis Taxa, Paola Montenegro

**Affiliations:** 1 Departamento de Medicina Oncológica, Instituto Nacional de Enfermedades Neoplásicas, Lima 15038, Peru; 2 Escuela Profesional de Medicina Humana, Universidad Privada San Juan Bautista, Lima 15067, Peru; 3 Department of Gastrointestinal Medical Oncology, The University of Texas MD Anderson Cancer Center, Houston, TX 77030, USA; 4 Department of Medical Oncology, Hospital Ramón y Cajal, Madrid 28034, Spain; 5 Departamento de Cirugía en Abdomen, Instituto Nacional de Enfermedades Neoplásicas, Lima 15038, Peru; 6 Departamento de Patología, Instituto Nacional de Enfermedades Neoplásicas, Lima 15038, Peru

**Keywords:** gastric cancer, adjuvant chemotherapy, survival

## Abstract

**Background:**

Gastric cancer (GC) is the fourth most common cause of cancer deaths around the world and the first cause of cancer deaths in Peru; however, there are no prospective trials for adjuvant chemotherapy in GC after curative gastrectomy in this country. The objective of this study was to evaluate the effectiveness of adjuvant chemotherapy in stage II–III gastric cancer patients who underwent D2 gastrectomy.

**Methods:**

We included patients with stage II–III gastric cancer who underwent radical gastrectomy and D2 dissection between 2014 and 2016 at our institution. Patients received 3-week cycles of capecitabine (1,000 mg/m^2^ twice daily on days 1–14) plus oxaliplatin (130 mg/m^2^ on day 1) for 6 months. Survival curves were estimated with the Kaplan–Meier method, and the Cox proportional hazards model was used to identify prognostic factors for survival.

**Results:**

In total, 173 patients were included: 100 (57.8%) patients received adjuvant chemotherapy and surgery (AChS) and 73 (42.2%) surgery alone (SA). Three-year disease-free survival (DFS) was higher in the AChS groups (69%) than in the SA group (52.6%) (*p* = 0.034). Regarding overall survival (OS), 31 patients (31%) died in the AChS group compared with 34 (46.6%) in the SA group (*p* = 0.027). In the multivariate analysis, adjuvant chemotherapy was an independent prognostic factor for DFS (HR = 0.60; 95% CI = 0.37–0.97; *p* = 0.036) and OS (HR = 0.58; 95% CI = 0.36–0.95; *p* = 0.029). ACh showed consistent benefit in DFS and OS for patients with albumin >3.5 g/dL, lymphovascular and perineural invasion, pT4, pN2–3, pathologic stage (PS) IIIA and IIIB and lymph node ratio (LNR) > 13.1.

**Conclusion:**

These data suggest that adjuvant capecitabine and oxaliplatin reduce the recurrence and mortality in patients with stage II–III gastric cancer who underwent D2 gastrectomy. PS IIIA and IIIB and LNR > 13.1 benefited more from receiving adjuvant chemotherapy and poorly cohesive gastric carcinoma did not significantly reduce the rates of survival.

## Introduction

Gastric cancer (GC) is the fourth most common cause of cancer deaths around the world and the first cause of cancer deaths in Peru [1]. GC mostly affects older people with an average age at diagnosis around 68 years old. The risk to develop this cancer is about 1 in 95 for men and 1 in 154 for women [2, 3]. *Helicobacter pylori* is a known and important carcinogenic factor for gastric cancer in our country. According to pathological studies, it has been reported that 54.76% of gastric cancer patients had *H. pylori*; while with molecular evaluation it was found that 94% were positive for this bacterium [4]. Improved socio-economic status, hygienic practices and widespread antibiotic use have led to a decrease in infection rates [5].

The standard of care for localised gastric cancer includes surgical resection. However, there is no global consensus on the optimal treatment approach for gastric cancer due to the various surgical techniques and other practices used in different parts of the world. D2 lymph node dissection is additional removal of a second tier of lymph nodes in the extraperigastric areas, which generally fall along branches of the celiac axis, including the left gastric, splenic, common hepatic and proper hepatic arteries. This surgical technique is practiced commonly in Japan and Korea but is less common in other countries [6]. In eastern Asia, the standard approach is surgical resection with D2 lymphadenectomy, followed by adjuvant chemotherapy, whereas in Western countries there is a preference for using either perioperative chemotherapy or postoperative chemoradiation, especially in cases of inadequate (<D2) lymph node dissection [7]. In our country, adjuvant chemotherapy after D2 lymph node dissection is considered the standard treatment and the postoperative morbidity and mortality in D2 radical gastrectomy for gastric cancer in Peruvian patients are 23.3% and 3.3%, respectively [8].

The REGATE study was the largest international prospective registry that enrolled patients with newly diagnosed gastric cancer. In terms of adjuvant chemotherapy, the REGATE data indicated that this approach is most commonly used for stage III cancers in all regions and is more frequently practiced in the Asia-Pacific and Latin American regions than in Europe. Fluoropyrimidine-based adjuvant chemotherapy regimens are the most common globally but patients in the Asia-Pacific region are much more likely than those in other parts of the world to receive newer oral fluoropyrimidines [9].

The Japanese ACST-GC trial was the first large-scale randomised trial of adjuvant chemotherapy after curative resection with D2 gastrectomy with stage II–III gastric cancer that shows 80% of overall survival (OS) compared with surgery alone (70%) [10]. Also, the Korean Classic trial was the second largest trial of adjuvant chemotherapy after D2 gastrectomy, showing 78% in OS versus surgery alone (69%) [11]. In addition, the GASTRIC group meta-analysis suggests a 5.8% absolute OS benefit at 5 years (55.3%–49.6%) for patients treated with adjuvant chemotherapy [12].

There are no prospective trials for adjuvant chemotherapy in gastric cancer after curative gastrectomy in Peru. The objective of this study was to evaluate the effectiveness of adjuvant chemotherapy in the Peruvian population with stage II–III gastric cancer who underwent radical gastrectomy and D2 lymph node dissection, and identify prognostic factors of OS and DFS in patients treated with adjuvant chemotherapy.

## Materials and methods

### Study design

This was an observational and analytic study. We retrospectively reviewed data, obtained from the medical records of the National Institute of Neoplastic Diseases (INEN) in Lima-Peru, of patients diagnosed with gastric cancer between January 2014 and December 2016.

### Patients and eligibility criteria

We included patients aged 18 years or older with stage II–III gastric cancer who underwent radical gastrectomy and D2 dissection. In case of patients treated with adjuvant chemotherapy, inclusion criteria were six or more courses of capecitabine plus oxaliplatin; patients who received chemotherapy as follows: 3-week cycles of oral capecitabine (1,000 mg/m^2^ twice daily on days 1–14 of each cycle) plus intravenous oxaliplatin (130 mg/m^2^ on day 1 of each cycle).

Patients were ineligible if they received preoperative therapy, adjuvant radiotherapy or death by immediate postoperative complications.

### Demographic and clinical variables

Demographic data included age and gender. Neutrophil/lymphocyte ratio less than 5 [13], albumin values ≥3.5 g/dL [14] and albumin/globulin ratio greater than 1.5 [15] were considered normal values. Tumour location was evaluated by upper gastrointestinal endoscopy study and abdominal computed tomography with contrast. Lauren [16] and the WHO’s histological classifications [17] were determined by pathological anatomy of the surgical piece, as well as the evaluation of lymphovascular invasion, perineural invasion, degree of differentiation and lymph node ratio (LNR) (number of positive lymph nodes/total number of lymph nodes excised) [18]. The database also had TNM classification according to the American Joint Committee on Cancer, 8th edition [19].

### Statistical analysis

Differences according to the type of treatment received (surgery alone and surgery plus chemotherapy) were evaluated with the Mann–Whitney U test in quantitative variables (after evaluating the assumption of normality), while qualitative characteristics were evaluated with the chi-square test.

DFS was estimated from the date of surgery to the date of recurrence or the date of death or the date of the last control, and OS was estimated from the date of surgery to the date of death or the date of consultation of the patient’s vital condition in the National Identification Registry (RENIEC). Survival curves were estimated with the Kaplan–Meier method and the log-rank test was used to compare them. The Cox proportional hazards model was used to calculate hazard ratios and identify prognostic factors for survival.

A p-value < 0.05 was considered statistically significant and the analysis was carried out with R Studio Software (version 1.3.959; RStudio PBC, Boston, MA, USA).

### Ethics

This study was approved by the ethics research committee of INEN. The data obtained from the medical records were kept confidential.

## Results

### Demographics and clinical characteristics of the patients

Between January 2014 and December 2016, 2,817 patients with GC were registered at INEN. Of these, 1,541 (54.7%) had clinical stage II–III. From them, 1,325 were excluded according to the exclusion criteria described above. In total, 173 gastric cancer patients with stage II–III who underwent radical gastrectomy and D2 dissection were included in this study: 100 (57.8%) patients received adjuvant chemotherapy and surgery and 73 (42.2%) surgery alone. Table 1 shows demographics and clinical characteristics according to type of treatment.

In comparison with the surgery alone group, the average age of patients in the adjuvant chemotherapy plus surgery group was lower (64.5 (±13.7) versus 56.8 years (±12.4), respectively, (*p* < 0.001)). Also, the frequency of lymphovascular invasion (91%), lymph node ratio >13 (55%), nodal status (pN) 2–3 (75%) and pathologic stage III (79%) were higher in the adjuvant chemotherapy plus surgery group (*p* < 0.05). No statistical differences between the groups according to sex, neutrophil/lymphocyte ratio, albumin/globulin ratio, site of tumour, Lauren and OMS’ classification, perineural invasion, differentiation and tumour stage (pT) were observed (Table 1).

### Characteristics of adjuvant chemotherapy

The median of time to adjuvant chemotherapy since surgery was 6.64 weeks (range: 3.14–19.0). Regarding treatment duration, 34.0% (*n* = 34) of the patients received chemotherapy for 6 months or less; 46.0% (*n* = 46) from 6.1 to 8 months and 20% for more than 8 months. In total, 36.0% (*n* = 36) received six courses, 19% (*n* = 19) seven courses and 45% (*n* = 45) eight courses of chemotherapy.

### Adverse events

From patients treated with adjuvant chemotherapy, 85% (*n* = 85) experienced adverse effects. The most commonly reported adverse events at any grade in the chemotherapy group were neutropenia, nausea, peripheral neuropathy and diarrhoea. The most common grade 3 or 4 adverse events were neutropenia. Adverse events led to chemotherapy dose modifications in 72 (72.0%) patients; neutropenia, nausea and peripheral neuropathy were the most common reasons (Table 2).

### Disease-free and overall survival

The median follow-up for DFS was 46 months (45.17–47.62) and 48 months for OS (46.22–50.96).

**Figure 1.**
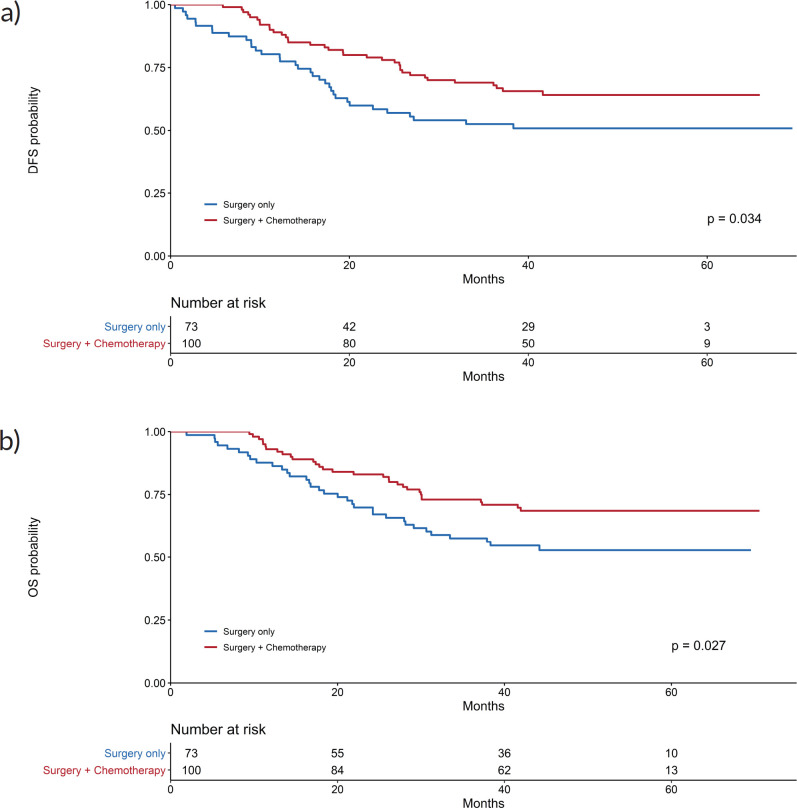
Kaplan–Meier curves for (a): DFS and (b): OS.

The 3-year disease-free survival was higher in the adjuvant chemotherapy and surgery group (69%) than in the surgery alone group (52.6%) (*p* = 0.034). Kaplan–Meier curves for disease-free survival show early separation between the two study groups (Figure 1a), although median disease-free survival was not reached.

Regarding OS, 31 patients (31%) died in the adjuvant chemotherapy group compared with 34 (46.6%) in the surgery only group (*p* = 0.027); the median OS was not reached (Figure 1b). Three-year OS was 73% in the adjuvant chemotherapy plus surgery group and 57.5% in the surgery alone group.

In the multivariate analysis, adjuvant chemotherapy was an independent prognostic factor for survival, for both DFS (HR = 0.28, 95% CI = 0.17–0.47; *p* < 0.001) and OS (HR = 0.27, 95% CI = 0.16–0.46; *p* < 0.001) (Tables 4 and 5).

### Prognostic factors for survival according to treatment

**Figure 2.**
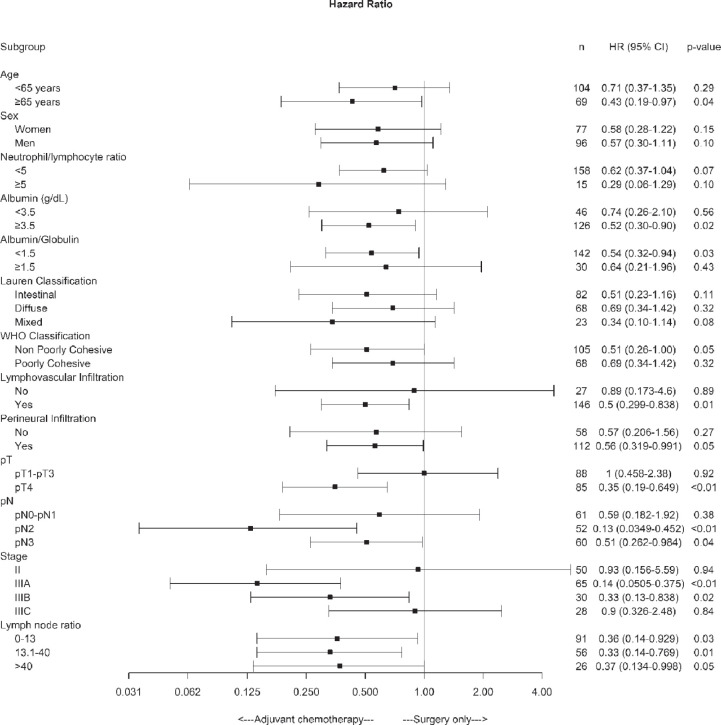
Forest plot of the treatment effect on disease-free survival in specific subgroups.

Subgroup analysis of disease-free survival showed consistent benefits for chemotherapy plus surgery compared with surgery alone for patients with albumin >3.5 g/dL, non-poorly cohesive subtypes by the WHO’s classification, lymphovascular and perineural invasion, pT4, pN2–3, pathologic stage IIIA and IIIB and LNR > 13.1 factors (Figure 2).

**Figure 3.**
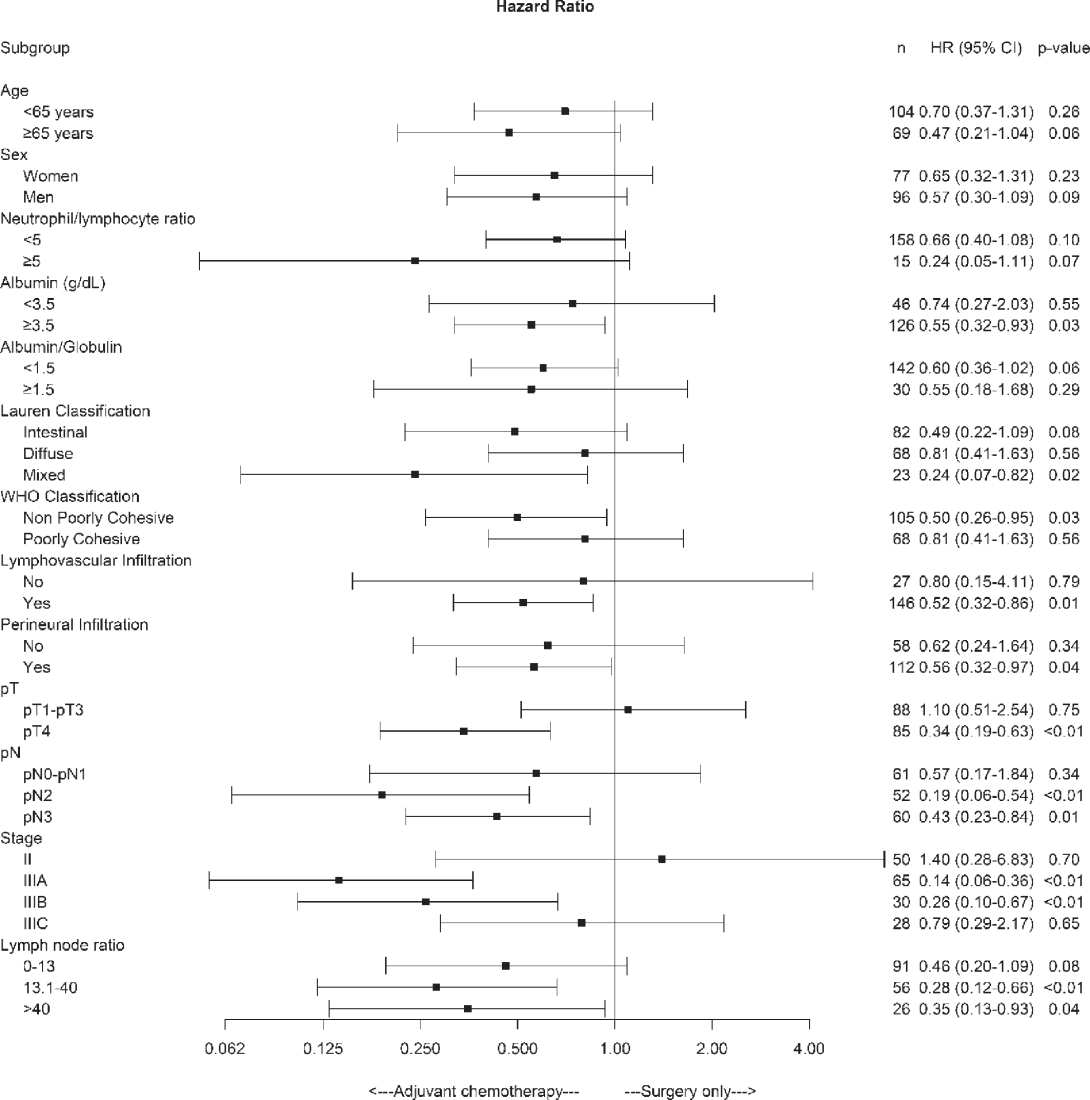
Forest plot of the treatment effect on overall survival in specific subgroups.

On the other hand, OS was significantly higher in the chemotherapy plus surgery group than in the surgery alone group for patients with albumin >3.5 g/dL, lymphovascular and perineural invasion, pT4, pN2–3, pathologic stage IIIA and IIIB and LNR > 13.1 factors (Figure 3).

## Discussion

In this study, we compared the effectiveness of adjuvant chemotherapy plus surgery versus only surgery in Peruvian patients with stage II–III gastric cancer who underwent radical gastrectomy and D2 lymph node dissection. Although patients in the AChs group were younger and had poor prognostic characteristics, the OS and DFS were longer in patients with gastric cancer who received adjuvant chemotherapy with CAPOX compared to those with only surgery treatment. In the multivariate analysis where possible confounders were included in the model, adjuvant chemotherapy was an independent prognostic factor for survival, with DFS (HR = 0.28, 95% CI = 0.17–0.47; *p* < 0.001) and OS (HR = 0.27, 95% CI = 0.16–0.46; *p* < 0.001). The decreased risk of death and recurrence was greater than those published in the GASTRIC group meta-analysis of 17 trials, which showed a reduction of 18% for both disease-free survival and OS in patients with resectable disease [12].

Furthermore, the 3-year DFS and OS were higher in the CAPOX adjuvant chemotherapy and surgery group than in the surgery alone group (69% versus 52.6%, *p* = 0.034; 31% versus 46.6%, *p* = 0.027, respectively). These results are similar to other studies that investigated postoperative adjuvant chemotherapy regimens after D2 lymph node dissection without radiotherapy or neoadjuvant therapy [10, 11]. In addition, the main guidelines of management of gastric cancer recommend a multidisciplinary approach in treatment planning, whether to use neoadjuvant or adjuvant CHT, both with clinical benefits in DFS and OS.

In subgroup analysis of disease-free survival and OS according to treatment, age >65 years showed benefits for chemotherapy plus surgery compared with surgery alone (HR = 0.46 *p* = 0.006 and HR = 0.43 *p* = 0.004, respectively). Some studies confirm these results, the adjuvant chemotherapy in elderly patients with gastric cancer has same effectiveness as non-elderly patients, with median survival rates around 20.8 months in patients younger than 65 years and 19.5 months in patients aged 65 years or older [20, 21]. Based on the available data, it seems clear that adjuvant chemotherapy is as effective in elderly patients with GC as younger patients if it is administered with more caution under careful monitoring for severe toxicities. However, many oncologists hesitate to recommend elderly patients to receive chemotherapy because comorbidities or age-related changes, pharmacokinetics and pharmacodynamics may lead to higher toxicity.

About 40% of our patients had poorly cohesive gastric cancer (PCGC), a distinct type of gastric cancer that is persistently increasing in Asia, Europe and the United States, and accounts for 35%–45% of new adenocarcinoma cases [22, 23]. PCGC is frequently and highly infiltrative and resistant to chemotherapy [24, 25]. Although radical gastrectomy is a standard treatment for PCGC, recurrence is a critical issue for long-term survival of patients, and so far, the role of the adjuvant chemotherapy in these patients is controversial [26]. Our data suggest that adjuvant chemotherapy did not significantly reduce DFS (51.7% versus 43.6%, surgery alone and surgery plus adjuvant chemotherapy group, respectively) and OS (51.7% versus 38.5%, surgery alone and surgery plus adjuvant chemotherapy group, respectively) after curative resection and D2 lymph node dissection in poorly cohesive gastric carcinoma. Therefore, understanding its molecular mechanisms and effective therapeutic options remains a challenge.

LNR remains as an important independent prognostic factor in patients undergoing radical gastrectomy and D2 lymph node dissection for gastric cancer, despite the various cut-off points used in its classification [18, 27, 28]. In our study, a subgroup analysis of disease-free survival and OS showed consistent benefits for chemotherapy plus surgery compared with surgery alone for lymph node ratio of 13.1 to >40, which is a good finding because different studies have found that this group of patients has a poor prognosis. Guevara *et al* [29] found LNR as an important prognostic factor to explain the time of death (LNR = 13.1–40; HR = 6.77; 95% CI = 3.34–13.70, *p* < 0.05) and recurrence time (LNR = 13.1–40; 95% CI = 2.10–13.43; *p* < 0.05) in gastric cancer patients who underwent radical gastrectomy D2 treated at INEN [29]. Likewise, Kim *et al* [30] have demonstrated that adjuvant treatment in patients with D2 lymphadenectomy and with a high LNR (≥0.25) had greater benefit with better disease-free survival compared to those who did not receive.

Several studies have noted that postoperative recurrence is associated with factors such as pT, extent of lymph node invasion and clinical stage [31–34], which is consistent with this study. The majority of our patients were diagnosed as advanced gastric cancer, 50% as pT4, more than half as pN2/N3 and 65% as IIIA/IIIB clinical stage. For these patient groups, disease-free survival and OS were significantly improved with oxaliplatin and capecitabine after curative surgery compared with surgery only. These data are consistent with that reported in the CLASSIC trial [11].

This study has some limitations. The information was obtained retrospectively from the review of medical records and, in some cases, there were missing data. Likewise, the time of follow-up, although long, was insufficient since the median OS had not yet been reached. However, the source used is representative and allows to characterise our reality. Our future direction is to carry out a prospective and comparative analysis with others types of systemic treatment.

## Conclusion

These data suggest that adjuvant capecitabine and oxaliplatin reduce the recurrence and mortality in patients with stage II–III gastric cancer who underwent radical gastrectomy and D2 dissection. Patients with pathologic stage IIIA and IIIB and lymph node ratio >13.1 benefited more from receiving adjuvant chemotherapy. The addition of adjuvant chemotherapy in patients with poorly cohesive gastric carcinoma did not significantly reduce the rate of recurrence and mortality after D2 gastrectomy. However, prospective studies are required to confirm these findings.

## Conflict of interest

The authors declare that they have no conflict of interest.

## Funding

The authors received no financial support for the research, authorship, and/or publication of this article.

## Figures and Tables

**Table 1.** Patients’ demographics and clinical characteristics according to treatment.

	Total (%) 173 (100.0)	Surgery alone *n* (%) 73 (42.2)	Adjuvant chemotherapy plus surgery *n* (%) 100 (57.8)	*p*
**Age, years** **Media (standard deviation)**	60.0 (13.5)	64.5 (13.7)	56.8 (12.4)	**<0.001**
<40	13 (7.5)	4 (5.5)	9 (9.0)	**0.026**
41–65	97 (56.1)	34 (46.6)	63 (63.0)	
> 65	63 (36.4)	35 (47.9)	28 (28.0)	
**Sex**				0.163
Women	77 (44.5)	37 (50.7)	40 (40.0)	
Men	96 (55.5)	36 (49.3)	60 (60.0)	
**Neutrophil/lymphocyte ratio**				0.467
<5	158 (91.3)	68 (93.2)	90 (90.0)	
≥5	15 (8.7)	5 (6.8)	10 (10.0)	
**Albumin (g/dL)** **Media (standard deviation)**	3.8 (0.6)	3.7 (0.6)	3.8 (0.6)	0.363
<3.5	46 (26.7)	20 (27.8)	26 (26.0)	0.795
≥ 3.5	126 (73.3)	52 (72.2)	74 (74.0)	
Unknown	1			
**Albumin/globulin**				
<1.5	142 (82.6)	63 (87.5)	79 (79.0)	0.147
≥1.5	30 (17.4)	9 (12.5)	21 (21.0)	
Unknown	1			
**Site of tumour**				0.169
Antrum	116 (67.0)	48 (65.7)	68 (68.0)	
Body	31 (17.9)	15 (20.6)	16 (16.0)	
Body and antrum	19 (11.0)	7 (9.6)	12 (12.0)	
Fundus	3 (1.7)	3 (4.1)	0	
Fundus and body	3 (1.7)	0	3 (3.0)	
Whole gastric	1 (0.7)	0	1 (1.0)	
**Lauren classification**				0.450
Intestinal	82 (47.4)	37 (50.7)	45 (45.0)	
Diffuse	68 (39.3)	29 (39.7)	39 (39.0)	
Mixed	23 (13.3)	7 (9.6)	16 (16.0)	
**OMS classification**				0.892
Tubular adenocarcinoma	72 (41.6)	31 (42.5)	41 (41.0)	
Mixed adenocarcinoma	25 (14.5)	9 (12.3)	16 (16.0)	
Mucinous adenocarcinoma	8 (4.6)	4 (5.5)	4 (4.0)	
Poorly cohesive	68 (39.3)	29 (39.7)	39 (39.0)	
**Lymphovascular invasion**				**0.005**
Present	146 (84.4)	55 (75.3)	91 (91.0)	
Absent	27 (15.6)	18 (24.7)	9 (9.0)	
**Perineural invasion**				0.075
Present	112 (65.9)	42 (58.3)	70 (71.4)	
Absent	58 (34.1)	30 (41.7)	28 (28.6)	
Unknown	3			
**Differentiation**				0.402
Well	9 (5.2)	2 (2.7)	7 (7.0)	
Moderate	61 (35.3)	28 (38.4)	33 (33.0)	
Poor and undifferentiated	103 (59.5)	43 (58.9)	60 (70.0)	
**Lymph node ratio**				**0.002**
0	24 (13.9)	18 (24.7)	6 (6.0)	
0.1–13	67 (38.7)	28 (38.4)	39 (39.0)	
13.1–40	56 (32.4)	21 (28.8)	35 (35.0)	
>40	26 (15.0)	6 (8.2)	20 (20.0)	
**Tumour stage**				0.839
T1–T2	13 (7.5)	6 (8.2)	7 (7.0)	
T3	75 (43.4)	33 (45.2)	42 (42.0)	
T4	85 (49.1)	34 (46.6)	51 (51.0)	
**Nodal stage**				**0.003**
N0	24 (13.9)	18 (24.7)	6 (6.0)	
N1	37 (21.4)	18 (24.7)	19 (19.0)	
N2	52 (30.1)	20 (27.4)	32 (32.0)	
N3a	33 (19.1)	10 (13.7)	23 (23.0)	
N3b	27 (15.6)	7 (9.6)	20 (20.0)	
**Pathologic stage**				**0.002**
IIA	18 (10.2)	15 (20.5)	3 (3.0)	
IIB	32 (18.8)	14 (19.2)	18 (18.0)	
IIIA	65 (36.9)	27 (37.0)	38 (38.0)	
IIIB	30 (17.6)	10 (13.7)	20 (20.0)	
IIIC	28 (16.5)	7 (9.6)	21 (21.0)	

Significant *p*-values (<0.05) are shown in bold

**Table 2.** Most common adverse events (>5%) reported by patients treated with adjuvant chemotherapy (*n* = 85).

Adverse events	All grades	Grade 1 or 2	Grade 3 or 4
Neutropenia	36 (42.4%)	21 (24.7%)	15 (17.7%)
Nausea	34 (40%)	34 (40%)	0
Peripheral neuropathy	30 (35.3%)	29 (34.1%)	1 (1.2%)
Diarrhoea	27 (31.8%)	27 (31.8%)	0
Asthenia	11 (12.9%)	11 (12.9%)	0
Hand–foot syndrome	9 (10.6%)	9 (10.6%)	0
Thrombocytopenia	6 (7%)	5(5.8%)	1 (1.2%)
Anaemia	6 (7.1%)	6 (7.1%)	0

**Table 3.** Prognostic factor for DFS, univariate and multivariate analyses.

Characteristics	Univariate			Multivariate		
	HR	HR 95%	*p*-value	HR	HR 95%	*p*-value
**Age**						
<65	1					
≥65	1.04	0.64–1.68	0.881			
**Sex**						
Women	1					
Men	0.933	0.58–1.50	0.773			
**NF_ratio**						
<5	1					
≥5	1.28	0.59–2.80	0.535			
**Albumin (g/dL)**						
<3.5	1					
≥3.5	1.24	0.70–2.21	0.454			
**Albumin/Globulin**						
<1.5	1					
≥1.5	1.09	0.60–2.00	0.775			
**Lauren**						
Intestinal	1			1		
Diffuse	1.83	1.09–3.10	**0.023**	1.59	0.87–2.89	0.129
Mixed	2.04	1.02–4.07	**0.043**	1.3	0.60–2.80	0.504
**Who**						
Non Poorly cohesive	1					
Poorly cohesive	1.53	0.95–2.45	0.079			
**Lymphovascular invasion**						
No	1					
Yes	1.73	0.792–3.78	0.169			
**Perineural invasion**						
No	1			1		
Yes	1.75	1.01–3.03	**0.046**	1.11	0.59–2.07	0.752
**pT**						
pT1–pT3	1			1		
pT4	2.32	1.42–3.79	**0.001**	1.43	0.82–2.48	0.208
**pN**						
pN0–pN1	1			1		
pN2	1.57	0.753–3.26	0.230	0.98	0.36–2.65	0.970
pN3	4.36	2.32–8.17	**<0.001**	1.33	0.20–8.69	0.766
**Clinical stage**						
II	1			1		
IIIA	3.64	1.48–8.93	**0.005**	5.01	1.64–15.26	**0.005**
IIIB	8.49	3.38–21.3	**<0.001**	14.23	2.28–88.87	**0.005**
IIIC	9.75	3.92–24.2	**<0.001**	12.33	1.81–83.92	**0.010**
**Lymph node ratio**						
0–13	1					
13.1–40	1.77	0.987–3.18	0.055	0.51	0.18–1.45	0.206
>40	5.73	3.2–10.2	**<0.001**	1.23	0.36–4.25	0.740
**Treatment**						
Surgery	1			1		
Adjuvant chemotherapy	0.603	0.376–0.968	**0.036**	0.28	0.17–0.47	**<0.001**

Significant *p*-values (<0.05) are shown in bold

**Table 4.** Prognostic factor for OS, univariate and multivariate analyses.

Characteristics	Univariate			Multivariate		
	HR	HR 95%	*p*-value	HR	HR 95%	*p*-value
**Age**						
<65	1					
≥65	1.1	0.67–1.81	0.696			
**Sex**						
Women	1					
Men	0.978	0.60–1.60	0.930			
**NF_ratio**						
<5	1					
≥5	1.41	0.64–3.09	0.392			
**Albumin (g/dL)**						
<3.5	1					
≥3.5	1.29	0.71–2.33	0.400			
**Albumin/Globulin**						
<1.5	1					
≥1.5	1.17	0.64–2.14	0.619			
**Lauren**						
Intestinal	1			1		
Diffuse	1.82	1.07–3.12	**0.029**	1.92	1.08–3.43	**0.027**
Mixed	2.04	1.00–4.18	0.050	1.51	0.71–3.23	0.283
**Who**						
Non Poorly cohesive	1					
Poorly cohesive	1.53	0.94–2.49	0.088			
**Lymphovascular invasion**						
No	1					
Yes	1.62	0.74–3.54	0.231			
**Perineural invasion**						
No	1					
Yes	1.7	0.97–3.00	0.065			
**pT**						
pT1–pT3	1			1		
pT4	2.35	1.41–3.91	**0.001**	1.43	0.81–2.51	0.213
**pN**						
pN0–pN1	1			1		
pN2	1.31	0.61–2.78	0.488	0.70	0.26–1.88	0.475
pN3	4.05	2.15–7.63	**<0.001**	0.93	0.12–7.18	0.943
**Clinical stage**						
II	1			1		
IIIA	3.95	1.50–10.4	**0.006**	6.10	1.90–19.56	**0.002**
IIIB	9.12	3.38–24.6	**<0.001**	15.73	2.11–117.34	**0.007**
IIIC	11	4.11–29.4	**<0.001**	15.81	1.96–127.25	**0.009**
**Lymph node ratio**						
0–13	1			1		
13.1–40	1.95	1.07–3.55	**0.029**	0.63	0.21–1.83	0.393
>40	5.94	3.24–10.9	<0.001	1.59	0.46–5.50	0.467
**Treatment**						
Surgery	1			1		
Adjuvant chemotherapy	0.581	0.36–0.95	**0.029**	0.27	0.16–0.46	**<0.001**

Significant *p*-values (<0.05) are shown in bold
